# Effects of *Lactiplantibacillus plantarum* A458 on Lactose Intolerance in Mice: Associations with Gut Microbiota and Redox Balance

**DOI:** 10.3390/microorganisms14061273

**Published:** 2026-06-05

**Authors:** Rentao Zhang, Narandalai Danshiitsoodol, Masafumi Noda, Masanori Sugiyama

**Affiliations:** Department of Probiotic Science for Preventive Medicine, Graduate School of Biomedical and Health Sciences, Hiroshima University, Hiroshima 734-8551, Japan; d225530@hiroshima-u.ac.jp (R.Z.); bel@hiroshima-u.ac.jp (M.N.)

**Keywords:** Caco-2 cells, gut microbiota, *Lactiplantibacillus plantarum* A458, lactose intolerance, oxidative stress

## Abstract

Lactose intolerance (LI) is a common gastrointestinal disorder caused by reduced intestinal lactase activity, resulting in lactose maldigestion and digestive symptoms, including diarrhea, bloating, and abdominal pain. Supplementation with β-galactosidase-producing probiotics is a potential strategy to improve lactose metabolism and reduce symptoms. In this study, we evaluated the probiotic properties and LI-alleviating efficacy of *Lactiplantibacillus (L.) plantarum* A458 through integrated in vitro and in vivo analyses. The strain displayed robust survival under simulated gastric and bile environments, maintaining high viability at acidic pH (2.0) and in 0.3–0.5% bile salts. It also demonstrated active fermentation capacity, producing high levels of lactic acid, and showed strong adhesion to Caco-2 cells while effectively inhibiting the invasion of *Listeria monocytogenes*. In a mouse model of LI, oral administration of *L. plantarum* A458 reduced systemic inflammation, as evidenced by decreased serum levels of IL-1β, TNF-α, IL-6, and IFN-γ. Additionally, *L. plantarum* A458 improved hepatic antioxidant defenses by elevating SOD activity and reducing lipid peroxidation and redox imbalance, as indicated by decreased MDA levels and increased GSH/GSSG ratio. Notably, *L. plantarum* A458 increased jejunal lactase activity. Moreover, 16S rRNA sequencing suggested that *L. plantarum* A458 modulated the gut microbial community, with higher relative abundance of beneficial taxa and lower relative abundance of potentially pathogenic microbes. Collectively, these findings suggest that *L. plantarum* A458 shows potential as a probiotic candidate for managing LI, associated with increased lactase activity, reduced inflammation and oxidative stress, and modulated gut microbiota.

## 1. Introduction

Lactose intolerance (LI) is a prevalent digestive disorder primarily caused by insufficient lactase activity in the small intestine. Clinically, LI presents with a spectrum of gastrointestinal symptoms—including diarrhea, bloating, and abdominal pain—that impair dietary tolerance and substantially reduce quality of life [1,2]. Epidemiological data indicate that approximately 65–75% of adults worldwide experience varying degrees of lactose maldigestion [1], and the condition represents a significant individual and public health burden, particularly in Asian and African populations, where prevalence is highest [3].

To alleviate symptoms, individuals with LI frequently limit or avoid dairy consumption. However, such long-term dietary restrictions can inadvertently lead to deficiencies in essential nutrients [4], including calcium, vitamin D, and riboflavin, thereby potentially increasing the risk of osteoporosis and other health complications [5,6]. Current management strategies rely on lactose-free products or exogenous lactase supplementation [6,7]; yet their effectiveness is often inconsistent, as enzyme activity may vary widely depending on dosage, food matrix interactions, and interindividual physiological differences [8]. These limitations highlight the need for safe, effective, and sustainable biological approaches to enhance lactose digestion and restore intestinal homeostasis.

In recent years, probiotics have gained considerable attention as potential functional candidates for LI management [9,10,11]. Mechanistically, many probiotic strains possess intrinsic β-galactosidase activity that facilitates lactose hydrolysis and reduces the osmotic load associated with undigested lactose in the colon [6,12]. Beyond enzymatic assistance, probiotics contribute to gut health by modulating microbial community structure and SCFA-related metabolic functions, as well as reinforcing intestinal barrier integrity [13,14]. Notably, certain lactic acid bacteria (LAB) exhibit robust acid and bile tolerance, facilitating transient colonization or interaction with the intestinal mucosa and competitive inhibition of pathogenic adhesion [15]. LAB also exert immunomodulatory effects, attenuating proinflammatory cytokines (e.g., TNF-α, IL-6) and enhancing mucosal antioxidant defenses, thereby counteracting lactose-induced inflammation and oxidative stress [16,17].

Despite accumulating evidence supporting the functional benefits of LAB, comprehensive investigations that integrate in vitro probiotic characterization with in vivo validation in LI models remain limited. We isolated LAB strains from edible plants (*Wasabia japonica*) and selected *Lactiplantibacillus* (*L*.) *plantarum* A458 as a representative candidate for systematic evaluation. The primary aim of this study was to evaluate the probiotic potential of *L*. *plantarum* A458 in alleviating LI and to explore its effects on gut microbiota composition and redox balance in a mouse model. Accordingly, we assessed its acid and bile resistance, metabolic activity, and epithelial adhesion in vitro, and examined its ability to modulate inflammation, oxidative stress, and gut microbiota in vivo. This study provides additional insights into the functional potential of *L. plantarum* A458 and offers experimental evidence supporting probiotic-based strategies for LI management.

## 2. Materials and Methods

### 2.1. Strain and Cell Culture Conditions

*L. plantarum* A458 was isolated from *Wasabia japonica* and maintained in our laboratory culture collection. The strain was cultivated in a modified de Man, Rogosa, and Sharpe (MRS) broth, in which glucose was completely replaced by lactose (20 g/L) as the sole carbon source to evaluate its lactose-metabolizing capacity [18]. The remaining components of the modified MRS medium were identical to those of standard MRS.

*L. monocytogenes* was cultured in Luria–Bertani (LB) broth at 37 °C. Human colorectal adenocarcinoma Caco-2 cells (RBRC, RCB2095) were obtained from the RIKEN BRC Cell Bank (Tsukuba, Japan) and cultured in high-glucose Dulbecco’s Modified Eagle’s Medium (DMEM; FUJIFILM Wako Pure Chemical Co., Osaka, Japan) supplemented with 10% fetal bovine serum (FBS, Life Technologies, Waltham, MA, USA) and 1% penicillin–streptomycin. Cells were maintained at 37 °C in a humidified atmosphere containing 5% CO_2_.

### 2.2. In Vitro Physiological Tolerance and Growth Characteristics

#### 2.2.1. Acid and Bile Salt Tolerance

Acid and bile salt tolerance were evaluated according to the method described by Charteris et al. [19], with slight modifications. Overnight cultures were adjusted to an optical density at 600 nm (OD600) of 1.0 and inoculated into modified MRS broth adjusted to pH 2.0 for acid tolerance testing or modified MRS broth supplemented with 0.3% or 0.5% (*w*/*v*) bile salts (FUJIFILM, Osaka, Japan) for bile tolerance assessment. Cultures were incubated at 37 °C under static conditions and sampled at designated time points (acid tolerance: 0 and 3 h; bile tolerance: 0 and 5 h). Viable cell counts (CFU/mL) were determined by plate counting on MRS agar. Survival rate (%) was calculated according to Equation (1):

(1) $$Survival\;rate\;(\%)={CFU}_{1}∕{CFU}_{0}\times 100$$

where ${CFU}_{0}$ and ${CFU}_{1}$ represent viable counts at 0 h and after incubation, respectively.

#### 2.2.2. Growth Characteristics and Organic Acid Production

Overnight cultures were inoculated (1%, *v*/*v*) in modified MRS containing lactose or glucose as a carbon source and incubated at 37 °C under static conditions. OD600 measurements were used for relative growth comparison, with L-lactic acid and pH data serving as complementary indicators. OD600 was measured at predetermined time intervals to construct growth curves. Culture pH was monitored simultaneously during incubation. After 24 h, culture supernatants were obtained by centrifugation (5000× *g*, 10 min, 4 °C), and L-lactic acid concentrations were determined using a commercial enzymatic assay kit based on lactate dehydrogenase (LDH; FUJIFILM Wako Pure Chemical Co., Osaka, Japan) according to the manufacturer’s instructions.

### 2.3. Caco-2 Cell Adhesion and Invasion Assays

#### 2.3.1. Adhesion and Invasion Assays

Adhesion and invasion assays were performed as previously described by Moroni et al. [20], with slight modifications. Briefly, activated bacterial cells and *L. monocytogenes* were harvested by centrifugation, washed twice with sterile PBS, and resuspended in PBS. Caco-2 cell monolayers were co-incubated with bacteria at a multiplicity of infection (MOI) of 10:1 (bacteria:cell) at 37 °C in a humidified atmosphere containing 5% CO_2_.

For the adhesion assay, after 1 h of incubation, monolayers were washed three times with PBS to remove non-adherent bacteria and lysed with 0.1% Triton X-100 in PBS. For the invasion assay, after 2 h of co-incubation, monolayers were washed with PBS and incubated with fresh DMEM containing gentamicin (100 μg/mL; FUJIFILM Wako Pure Chemical Co., Osaka, Japan) for 1 h to eliminate extracellular bacteria, followed by cell lysis as described above. Cell lysates were serially diluted and plated on MRS or LB agar plates for colony-forming unit (CFU) enumeration.

Adhesion or invasion rate (%) was calculated according to Equation (2):

(2) $$Rate\;(\%)=CFU_{1}∕CFU_{0}\times 100$$

where $CFU_{1}$ represents the number of adherent or intracellular bacteria recovered from lysed cells, and $CFU_{0}$ represents the total number of bacteria in the initial inoculum.

#### 2.3.2. Inhibition of *L. monocytogenes* Invasion by *L. plantarum* A458

To evaluate the inhibitory effect of *L. plantarum* A458 on *L. monocytogenes* invasion, Caco-2 cells were co-incubated with *L. plantarum* A458 and *L. monocytogenes* at a multiplicity of infection (MOI) of 10:1 for 2 h at 37 °C. After incubation, monolayers were washed with PBS to remove non-adherent bacteria and then treated with gentamicin (100 μg/mL) for 1 h to eliminate extracellular bacteria. Subsequently, cells were lysed, and intracellular bacteria were enumerated by plating serial dilutions on LB agar plates for colony-forming unit (CFU) counting.

The competitive invasion rate was calculated as Equation (3):

(3) $$Competitive\;invasion\;rate\;(\%)=CFU_{1}/CFU_{0}\times 100$$

where $CFU_{1}$ represents the number of invaded bacteria recovered from lysed cells, and $CFU_{0}$ represents the total number of bacteria in the initial inoculum. The inhibitory effect of *L. plantarum* A458 on *L. monocytogenes* invasion was expressed as the percentage reduction relative to the *L. monocytogenes*-only group.

### 2.4. Mouse Model of LI and Intervention

All animal experiments were conducted in accordance with the Guidelines for the Care and Use of Laboratory Animals of Hiroshima University and were approved by the Institutional Animal Care and Use Committee (Approval No. A25-196). Eight-week-old male BALB/c mice (specific-pathogen-free, SPF; Shimizu Laboratory Supplies Co., Ltd., Kyoto, Japan) were housed under controlled conditions (temperature 20–26 °C, humidity 40–60%, 12 h light/dark cycle) with free access to standard chow and water.

After one week of acclimatization, mice were randomly divided into three groups (n = 5): healthy control (NC), lactose intolerance (LI), and *L. plantarum* A458 (LP). The experimental design consisted of three phases. During the prevention phase (Week 1), the LP group received daily oral gavage of 200 μL bacterial suspension (1 × 10^9^ CFU/mouse) for 7 days, while the NC and LI groups received an equal volume of sterile saline. During the induction phase (Week 2), the LI and LP groups were provided with 30% (*w*/*v*) lactose solution as drinking water to induce LI, whereas the NC group continued to receive normal drinking water. Probiotic administration was paused during the induction phase. The lactose concentration was selected based on previous studies demonstrating reliable induction of lactose maldigestion-related gastrointestinal symptoms in rodents [21,22,23,24]. To confirm successful establishment of the LI model, we evaluated the following parameters: body weight changes (daily recording), and clinical manifestations (diarrhea, fecal water content, fur condition, and spontaneous activity) at the end of the induction phase. During the intervention phase (Week 3), the LP group continued to receive oral gavage of 200 μL bacterial suspension (1 × 10^9^ CFU/mouse/day), and the NC and LI groups were administered saline.

Body weight was recorded daily throughout the experiment. At the end of the study, mice were euthanized under isoflurane anesthesia, and blood samples were collected via the abdominal aorta. Blood samples were centrifuged at 4000× *g* for 10 min at 4 °C to obtain serum, which was stored at −80 °C. Jejunum and corresponding intestinal contents, as well as liver tissues, were rapidly collected, snap-frozen in liquid nitrogen, and stored at −80 °C for further analysis.

### 2.5. Measurement of Serum Inflammatory Cytokines

Serum cytokines—including IFN-γ (Cat: 430801), IL-6 (Cat: 431301), TNF-α (Cat: 430901), and IL-1β (Cat: 432601)—were quantified using BioLegend ELISA kits (San Diego, CA, USA) according to the manufacturer’s protocols. Briefly, 100 μL of standards or serum samples were added to antibody-coated 96-well plates and incubated at room temperature. After washing, enzyme conjugates, substrates, and stop solution were sequentially added. The optical density was measured at 450 nm using a microplate reader (Varioskan, Thermo Scientific, Vantaa, Finland), and cytokine concentrations were calculated based on standard curves. All samples were analyzed in duplicate.

### 2.6. Determination of Hepatic Oxidative Stress Parameters

Liver tissues were homogenized on ice at a ratio of 10 mg tissue per 100 μL of the appropriate assay buffer for the determination of superoxide dismutase (SOD) activity, malondialdehyde (MDA) content, and the reduced-to-oxidized glutathione ratio (GSH/GSSG). For SOD measurement, tissues were homogenized in sucrose buffer and centrifuged at 10,000× *g* for 60 min at 4 °C according to the manufacturer’s instructions. For MDA determination, samples were homogenized in the assay buffer provided with the kit and centrifuged at 10,000× *g* for 5 min. For GSH/GSSG analysis, tissues were homogenized in 5% sulfosalicylic acid solution and centrifuged at 8000× *g* for 10 min at 4 °C.

The resulting supernatants were analyzed using commercial assay kits, including a WST-based SOD Assay Kit (Code: S311, Dojindo, Kumamoto, Japan), an MDA Assay Kit (Code: M496, Dojindo, Japan), and a GSH/GSSG Ratio Assay Kit (Code: G257, Dojindo, Japan). Protein concentrations were determined using the bicinchoninic acid (BCA) method with bovine serum albumin as the standard. Results were expressed as U/mg protein for SOD activity, nmol/mg protein for MDA content, and the GSH/GSSG ratio.

### 2.7. Determination of Intestinal Lactase Activity

Jejunum tissues were rinsed with ice-cold saline, blotted dry, and homogenized in saline (1:9, *w*/*v*), followed by centrifugation at 10,000× *g* for 20 min at 4 °C to collect the supernatants. Jejunal β-galactosidase (lactase) activity was determined using o-nitrophenyl-β-D-galactopyranoside (ONPG) as the substrate. The supernatants were incubated with ONPG at 37 °C for 30 min under standard assay conditions, and the reaction was terminated by adding sodium carbonate stop solution prior to measuring absorbance at 420 nm. Lactase activity was calculated based on the amount of o-nitrophenol released per minute using a standard curve, normalized to protein concentration, and expressed as U/mg protein.

### 2.8. Gut Microbiota Analysis

Total genomic DNA was extracted using an enzymatic–chemical lysis method followed by phenol–chloroform extraction. Briefly, fecal samples were suspended in phosphate-buffered saline, filtered to remove debris, and centrifuged to obtain pellets, which were resuspended in TE10 buffer. Bacterial cells were lysed sequentially using lysozyme and achromopeptidase, followed by SDS and proteinase K treatment. Genomic DNA was extracted with phenol/chloroform/isoamyl alcohol, precipitated with isopropanol, washed with 75% ethanol, and dissolved in TE buffer. RNase A treatment and polyethylene glycol precipitation were performed to further purify the DNA.

DNA quality was assessed by agarose gel electrophoresis, and DNA concentration was measured using a Qubit fluorometer (Thermo Fisher Scientific, Waltham, MA, USA). Qualified DNA samples were subjected to amplification of the V4 region (515F/806R) of the bacterial 16S rRNA gene and sequenced on an Illumina NovaSeq 6000 platform (San Diego, CA, USA). After quality filtering and chimera removal, the average sequencing depth was 29,880 effective reads per sample (range 28,908–31,350), with Good’s coverage >99% for all samples. Raw paired-end sequencing reads were processed using QIIME2 (version 2024.2), including quality filtering, denoising, feature table construction, and taxonomic classification using the SILVA 138.1 database, followed by downstream diversity and community composition analyses. Due to the limited sample size, only descriptive comparisons of relative abundances were performed. Formal alpha/beta diversity analyses and differential abundance tests were not conducted, and the results are interpreted as preliminary and indicative.

### 2.9. Statistical Analysis

Statistical analyses were performed using GraphPad Prism (2020), version 9.0 (San Diego, CA, USA). In vitro experiments were conducted in triplicate, and data are presented as mean ± standard deviation (SD). Differences among multiple groups were analyzed using one-way analysis of variance (ANOVA) followed by Tukey’s multiple comparisons test. Data normality was assessed prior to ANOVA. Differences were considered statistically significant at * *p* < 0.05, ** *p* < 0.01, and *** *p* < 0.001.

## 3. Results

### 3.1. Acid and Bile Salt Tolerance and Metabolic Characteristics of L. plantarum A458

**Figure 1:**
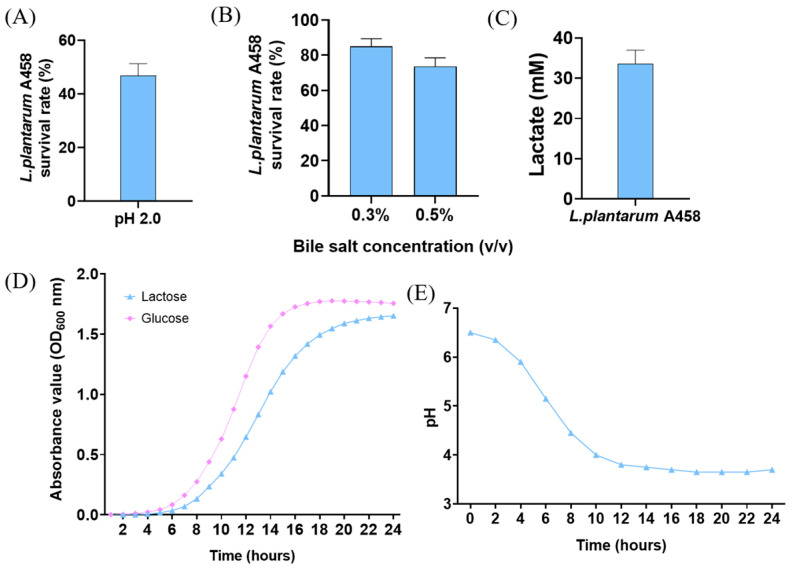
Gastrointestinal tolerance and metabolic characteristics of *L. plantarum* A458. (**A**) Survival of *L. plantarum* A458 after 3 h exposure to acidic conditions (pH 2.0). (**B**) Survival of *L. plantarum* A458 under different concentrations of bile salts. (**C**) Lactic acid production after 24 h cultivation. (**D**) Growth curves of *L. plantarum* A458 in modified MRS containing lactose or glucose as carbon source over 24 h. (**E**) Change in culture medium pH during 24 h cultivation.

The gastrointestinal tolerance of *L. plantarum* A458 was evaluated by assessing its resistance to acidic conditions and bile salts. As shown in Figure 1A, after 3 h exposure to pH 2.0, the strain exhibited a survival rate of approximately 47%. Under bile salt stress (Figure 1B), survival rates remained above 70% across all tested concentrations, reaching approximately 85% at 0.3% bile salt. As shown in Figure 1D, *L. plantarum* A458 reached stationary phase earlier (about 16 h) in glucose than in lactose (approximately 20 h), but achieved comparable maximum cell density in both carbon sources, confirming its ability to efficiently utilize lactose. After 24 h of cultivation, a marked decrease in medium pH was observed (Figure 1E), accompanied by lactic acid production of approximately 33 mM (Figure 1C).

### 3.2. Adhesion to Caco-2 Cells and Inhibition of L. monocytogenes Invasion

The interaction of *L. plantarum* A458 with intestinal epithelial cells was evaluated by assessing its adhesion capacity to Caco-2 cells and its antagonistic activity against *L. monocytogenes* invasion. As summarized in Table 1, *L. plantarum* A458 exhibited an adhesion rate of 7.47% to Caco-2 cells. Under the same experimental conditions, *L. monocytogenes* exhibited an invasion rate of 2.17%. Co-incubation with *L. plantarum* A458 significantly reduced the invasion of *L. monocytogenes*, resulting in an inhibition rate of 67.28%.

### 3.3. Establishment of an LI Mouse Model and Intervention Effects of L. plantarum A458

**Figure 2:**
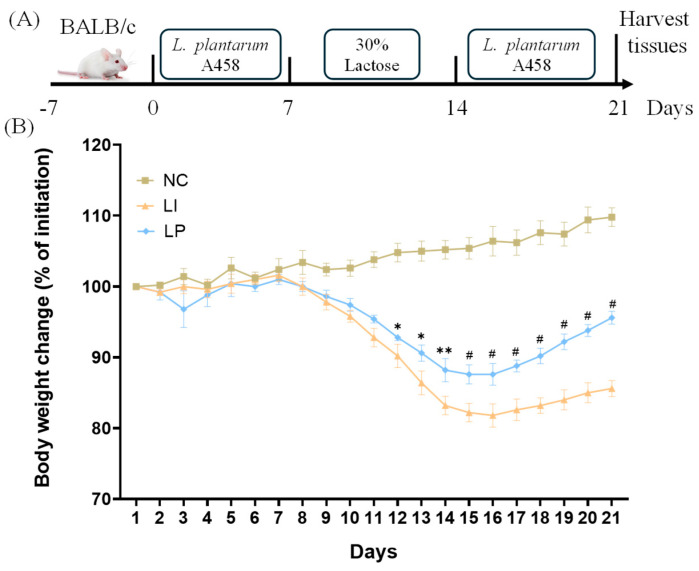
Experimental design and body weight changes in mice. (**A**) Experimental timeline of animal study. (**B**) Changes in body weight (%) relative to the initial body weight during the experimental period. * *p* < 0.05, ** *p* < 0.01, and ^#^ *p* < 0.001 as compared with LI group.

During the modeling phase (Figure 2A, experimental timeline), mice in the NC group exhibited no abnormal manifestations, whereas mice in the LI group gradually developed gastrointestinal symptoms consistent with lactose maldigestion, including diarrhea, increased fecal water content, roughened fur, and reduced spontaneous activity. Body weight analysis (Figure 2B) showed that mice in the LI group displayed a significant reduction in body weight compared with the NC group. In contrast, mice receiving oral administration of *L. plantarum* A458 prior to lactose challenge exhibited significantly lower body weight loss than the LI group, accompanied by improved fecal consistency. During the intervention phase, continued administration of *L. plantarum* A458 was associated with gradual improvement in stool appearance, with feces progressively transitioning from watery to formed. Although body weight in the LP group remained lower than that of the NC group during the early stage of intervention, it remained significantly higher than that of the LI group throughout the intervention period.

### 3.4. L. plantarum A458 Enhances Jejunal Lactase Activity

**Figure 3:**
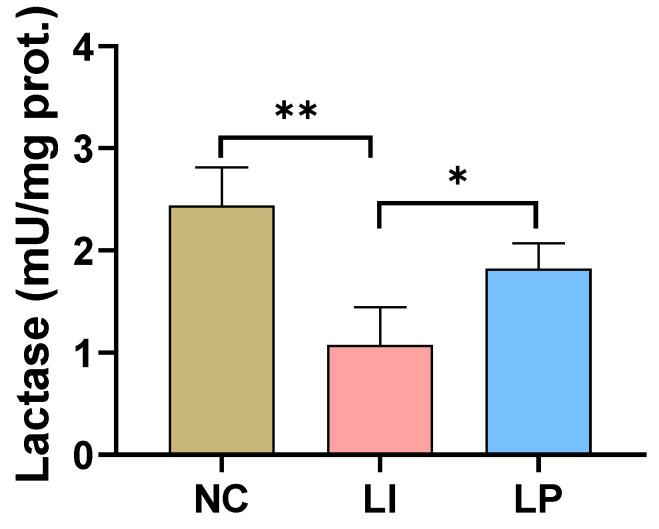
Effects of *L. plantarum* A458 on jejunal lactase activity in lactose-intolerant mice. Data are presented as mean ± SD. * *p* < 0.05, and ** *p* < 0.01 versus the LI group.

To evaluate intestinal lactose-digestive capacity, lactase activity in jejunal tissue was measured using an ONPG-based assay (Figure 3). Lactase activity in the LI group was significantly lower than that in the NC group. In contrast, administration of *L. plantarum* A458 significantly increased jejunal lactase activity compared with the LI group. The activity level in the LP group was intermediate between those of the NC and LI groups.

### 3.5. L. plantarum A458 Modulates Systemic Inflammatory Cytokine Levels

**Figure 4:**
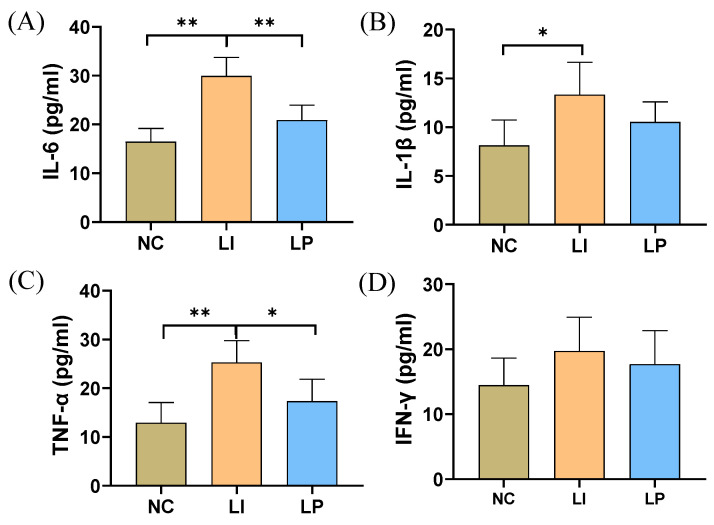
Effects of *L. plantarum* A458 on serum inflammatory cytokine levels in lactose-intolerant mice. (**A**) IL-6 in serum; (**B**) IL-1β in serum; (**C**) TNF-α in serum; (**D**) IFN-γ in serum. Data are presented as mean ± SD. * *p* < 0.05, and ** *p* < 0.01 versus the LI group.

Serum levels of interferon-γ (IFN-γ), interleukin-6 (IL-6), tumor necrosis factor-α (TNF-α), and interleukin-1β (IL-1β) were measured to evaluate systemic inflammatory status (Figure 4). Compared with the NC group, the LI group exhibited significantly increased serum levels of IL-6, TNF-α, and IL-1β, whereas IFN-γ showed an increasing trend without reaching statistical significance. Following administration of *L. plantarum* A458, serum IL-6 and TNF-α levels were significantly reduced compared with the LI group. In contrast, IL-1β and IFN-γ levels were lower than those in the LI group, although no statistically significant differences were observed.

### 3.6. Effects of L. plantarum A458 on Hepatic Oxidative Stress

**Figure 5:**
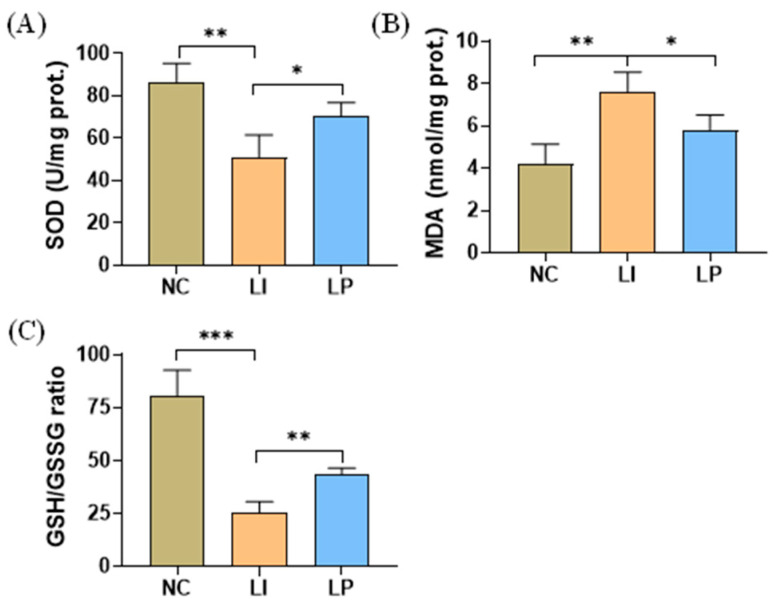
Effects of *L. plantarum* A458 on hepatic oxidative stress parameters in lactose-intolerant mice. (**A**) SOD activity in liver tissues. (**B**) MDA content in liver tissues. (**C**) GSH/GSSG ratio in liver tissues. Data are presented as mean ± SD. * p < 0.05, ** p < 0.01 and *** p < 0.001 versus the LI group.

Hepatic SOD activity, MDA content, and the GSH/GSSG ratio were measured to evaluate hepatic oxidative stress (Figure 5). Compared with the NC group, mice in the LI group exhibited significantly decreased SOD activity, significantly elevated MDA levels, and a markedly reduced GSH/GSSG ratio. Following administration of *L. plantarum* A458, hepatic SOD activity was significantly increased and MDA levels were significantly decreased compared with the LI group. On the other hand, the GSH/GSSG ratio was elevated in the LP group.

### 3.7. Effects of L. plantarum A458 on Jejunal and Ileal Microbiota Composition

**Figure 6:**
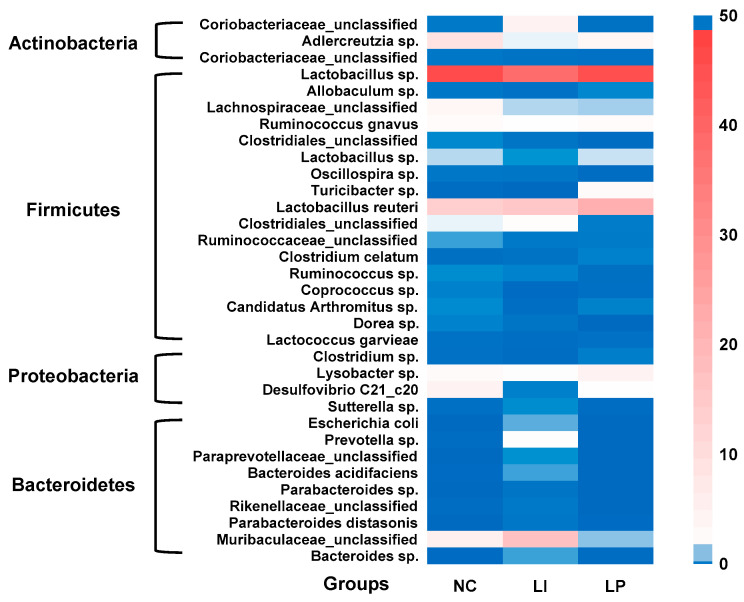
Heatmap showing the relative abundances of jejunal and ileal microbiota at the genus level following *L. plantarum* A458 intervention. Darker blue and red represent lower and higher relative abundances, respectively. No statistical tests were performed due to the small sample size.

Microbiota composition in the jejunal and ileal was analyzed by 16S rRNA high-throughput sequencing to evaluate the impact of oral administration of *L. plantarum* A458 (Figure 6). Heatmap analysis revealed distinct microbial distribution patterns among the NC, LI, and LP groups, suggesting marked differences in microbial composition.

As shown in the heatmap and relative abundance analysis, the LI group displayed distinct compositional shifts at the genus level compared with the NC group. Specifically, the relative abundances of *Lactobacillus*, *Allobaculum*, and *Turicibacter* were lower in the LI group than in the NC group, with *Turicibacter* not detected in relative terms. Following oral administration of *L. plantarum* A458, the levels of these genera increased to varying degrees. Moreover, *Lactobacillus*-related tax—including sequences affiliated with the *Lactiplantibacillus reuteri* phylogenetic cluster—showed higher abundance in the LP group compared with the LI group. Conversely, several genera (*Bacteroides*, *Prevotella*, *Parabacteroides*, *Escherichia*, and *Sutterella*) were more abundant in the LI group than in the NC group. After intervention with *L. plantarum* A458, the relative abundances of these taxa decreased, with some falling to levels close to or below the detection threshold. In addition, several *Ruminococcus*-related taxa were more abundant in the LI group and showed a decreasing trend following *L. plantarum* A458 administration, although their abundances did not fully return to the levels observed in the NC group.

## 4. Discussion

LI is a common digestive disorder characterized primarily by reduced lactose digestion capacity. In the present study, both in vivo and in vitro experiments were performed to assess the potential effects of *L. plantarum* A458 in an LI model. Under lactose challenge conditions, mice exhibited decreased lactase activity, elevated levels of inflammatory mediators, impaired antioxidant status, and noticeable alterations in gut microbiota composition compared with the NC group, reflecting multiple physiological changes associated with LI [22]. Administration of *L. plantarum* A458 was associated with partial improvement in these parameters, suggesting its possible involvement in the amelioration of LI-associated alterations.

In vitro assay showed that *L. plantarum* A458 maintained relatively high survival rates under simulated gastric acidic conditions (pH 2.0) and bile salt exposure, suggesting its capacity to tolerate gastrointestinal-like stress following oral administration. Adequate resistance to gastric acid and bile salts is generally considered an important prerequisite for probiotics to exert potential effects on gut microbiota and host physiology [25]. In addition, *L. plantarum* A458 exhibited notable acid-producing activity, with lactic acid identified as a major metabolic product. Organic acid production may contribute to environmental acidification, which has been reported to suppress the growth of certain opportunistic pathogens while favoring the proliferation of beneficial microorganisms such as LAB, thereby potentially contributing to microbial community balance [26]. Furthermore, adhesion assays showed that *L. plantarum* A458 adhered to Caco-2 cells and reduced *L. monocytogenes* invasion into intestinal epithelial cells. Its unconventional origin and distinctive multifunctional properties support the consideration of *L. plantarum* A458 as a potential LI probiotic candidate. Although in vitro adhesion and antagonistic activities cannot be directly extrapolated to in vivo colonization or functional outcomes, these findings provide preliminary evidence supporting the potential role of *L. plantarum* A458 in intestinal microbial interactions in vivo [27,28].

In the LI group, mice exhibited typical clinical manifestations, including body weight loss, diarrhea, and increased fecal water content, accompanied by a significant reduction in lactase activity, suggesting impaired intestinal lactose digestion capacity. Beyond these intestinal symptoms, the model mice also displayed evident systemic changes. Specifically, serum levels of several pro-inflammatory cytokines were significantly elevated, while hepatic antioxidant defense capacity was compromised, as reflected by decreased SOD activity, increased MDA levels, and a reduced GSH/GSSG ratio. Similar inflammatory and oxidative stress-related alterations have been reported in various models of LI and related gastrointestinal functional disorders, suggesting that lactose maldigestion may be accompanied by enhanced inflammatory responses and disturbed redox homeostasis at the systemic level [29,30]. It should be noted that these changes primarily represent concurrent physiological characteristics of the LI model rather than direct causal consequences of lactose maldigestion, and the underlying causal relationships remain to be further clarified.

From a conceptual perspective based on previous studies, inadequately hydrolyzed lactose reaching the colon has been proposed to promote the release of inflammatory mediators and to contribute to oxidative stress through abnormal fermentation processes, increased intestinal barrier permeability, and gut microbial dysbiosis, thereby potentially participating in the development of systemic inflammatory and oxidative responses [31,32]. This process has been proposed as a plausible explanation for the progression of LI from localized intestinal dysfunction to broader systemic alterations, rather than being solely attributable to lactase deficiency. Notably, oral administration of *L. plantarum* A458 correlated with improvements in multiple abnormal parameters, including increased lactase activity and reduced inflammatory cytokine levels. In addition, several antioxidant indices showed trends toward normalization compared with the NC group. These multi-level phenotypic improvements suggest that the effects of *L. plantarum* A458 may not be limited to enhanced lactose digestion alone but may also be associated with the modulation of inflammatory responses and oxidative stress, consistent with the alleviation of systemic physiological disturbances observed in the LI model. Nevertheless, the precise causal mechanisms underlying these effects remain to be further elucidated.

From a hypothetical mechanistic perspective based on previously reported probiotic functions, the potential effects of *L. plantarum* A458 may involve the coordinated contribution of multiple factors. On the one hand, its capacity for lactose fermentation and acid production may be associated with improved intestinal lactose digestion efficiency and modulation of the luminal microenvironment [33,34]. On the other hand, accumulating evidence suggests that LAB and their metabolites may exert anti-inflammatory and antioxidant effects through the modulation of inflammation- and oxidative stress-related signaling pathways, particularly NF-κB and Nrf2, although these pathways were not directly examined in the present study [35]. Previous studies have reported that *Lactobacillus*-derived probiotics can attenuate inflammatory responses through inhibition of NF-κB signaling while enhancing antioxidant gene expression via activation of the Nrf2 pathway, contributing to improved redox balance in various in vitro and in vivo models [36]. The reductions in pro-inflammatory cytokine levels and improvements in antioxidant indices observed in the present study are broadly consistent with these previously reported signaling modulation patterns rather than representing direct mechanistic evidence. Collectively, these observations raise the possibility that *L. plantarum* A458 could exert systemic protective effects through related signaling pathways; however, the precise molecular targets and mechanisms require further experimental validation.

The gut microbiota analysis provided additional microecological insights into the potential mechanisms by which *L. plantarum* A458 may be associated with alleviation of LI. It should be noted that 16S rRNA sequencing provides relative abundance data; thus, the observed alterations represent changes in the relative representation of taxa rather than absolute quantitative differences. Consequently, interpretations of microbiota modulation should be considered preliminary and indicative. High-throughput 16S rRNA sequencing indicated a pronounced disruption of gut microbial community structure in LI model mice, a lower relative abundance of certain taxa previously reported to be associated with intestinal homeostasis and metabolic functions, a higher relative abundance of several genera that have been linked to inflammatory or stress-associated intestinal environments. Previous studies suggest that such microbial dysbiosis may interfere with normal carbohydrate metabolism and potentially aggravate LI-related physiological disturbances by promoting inflammatory responses and impairing intestinal barrier integrity [37].

Following *L. plantarum* A458 intervention, the relative abundances of *Lactobacillus*, *Allobaculum*, and *Turicibacter* increased to varying degrees in relative terms. *Lactobacillus*, as a representative LAB, has been widely reported to possess β-galactosidase activity and to be associated with lactose metabolism, as well as with the maintenance of intestinal barrier homeostasis through modulation of the luminal microenvironment and competitive interactions with other microorganisms [38]. *Allobaculum* has been frequently linked to short-chain fatty acid production and host energy metabolism, and decreases in its relative abundance have been observed in various metabolic and inflammatory conditions [39]. *Turicibacter* has also been reported in microbiota-based studies to be associated with intestinal immune regulation and barrier function; however, its precise physiological roles remain incompletely understood [40]. The coordinated alterations observed in these taxa suggest that *L. plantarum* A458 intervention may be associated with a trend toward increased relative abundance of microbial communities related to metabolic balance and intestinal homeostasis.

In contrast, several genera, including *Bacteroides*, *Prevotella*, *Parabacteroides*, *Escherichia*, and *Sutterella*, showed a higher relative abundance in the LI group. Previous studies have reported that shifts in the relative abundance of these taxa may be linked to intestinal stress, microbial imbalance, increased inflammatory responses, or alterations in mucosal barrier function under specific pathological conditions [41]. In the present study, *L. plantarum* A458 administration was accompanied by decreases in the relative abundance of these genera. These microbial changes may reflect a tendency toward rebalancing of the intestinal microbial ecosystem; however, their functional implications should be interpreted with caution, as microbiota associations do not necessarily indicate direct causal relationships.

Taken together, these findings suggest that *L. plantarum* A458 intervention was associated with partial alleviation of LI-related alterations, accompanied by shifts in gut microbiota composition toward a profile more commonly linked to lactose metabolism and intestinal homeostasis, along with decreases in the relative abundance of genera that showed higher relative abundance under LI conditions. These microbial changes may reflect a trend toward increased relative abundance of the intestinal microbial ecosystem and a more balanced intestinal microenvironment. Similar patterns of coordinated changes involving microbiota modulation, attenuation of inflammatory responses, and improvements in metabolic-related indices have been described in previous probiotic intervention studies targeting LI and related intestinal functional disorders [42]. Notably, the observed microecological alterations were broadly consistent with reductions in inflammatory markers, improvements in antioxidant status, and increased jejunal lactase activity identified in the present study, with several indices showing trends toward improvement. From an application perspective, these findings suggest the possibility that *L. plantarum* A458 could be considered a candidate functional probiotic for dietary strategies aimed at managing LI-associated symptoms. Nevertheless, the present study has several limitations. The sample size (n = 5 per group) is relatively small, which may reduce statistical power and increase the risk of random variation or overestimation of the treatment effects. No dose–response assessment of *L. plantarum* A458 was performed, and no comparison with standard therapies was made. Consequently, the practical significance of the observed effects and whether the strain offers advantages over conventional approaches remain unknown. Furthermore, the study was limited to an animal model, and the underlying molecular mechanisms remain incompletely understood. Further investigations, including mechanistic studies, dose–response evaluations, and well-designed trials with larger cohorts and active comparators, are required to confirm its efficacy and safety in humans.

## 5. Conclusions

This study suggests a potential protective effect of *L. plantarum* A458 in a mouse model of LI, as indicated by good gastrointestinal tolerance, alleviation of lactose-induced diarrhea, increased jejunal lactase activity, reduced inflammatory and oxidative stress markers, and partial amelioration of gut microbiota dysbiosis. These changes suggest that *L. plantarum* A458 may be associated with maintenance of intestinal homeostasis through multiple physiological pathways. Collectively, this work provides preliminary experimental evidence supporting the potential of *L. plantarum* A458 as a candidate probiotic for the management of LI.

## Figures and Tables

**Table 1:** Adhesion capacity of *L. plantarum* A458 to Caco-2 cells and its inhibitory effect on the invasion of *L. monocytogenes*.

Bacterial Strain	Adhesion Rate (%)	Invasion Rate (%)	Competitive Invasion Rate (%)	Inhibition Rate (%)
*L. plantarum* A458	7.47 ± 0.77	-	-	-
*L. monocytogenes*	7.39 ± 0.99	2.17 ± 0.27	-	-
*L. plantarum* A458 and *L. monocytogenes*	-	-	0.71 ± 0.11 ***	67.28

Data are presented as mean ± SD (n = 3). *** *p* < 0.001 compared with *L. monocytogenes* invasion.

## Data Availability

The original contributions presented in this study are included in the article. Further inquiries can be directed to the corresponding authors.

## References

[1] Misselwitz B., Butter M., Verbeke K., Fox M.R. (2019). Update on lactose malabsorption and intolerance: Pathogenesis, diagnosis and clinical management. Gut.

[2] Costa J.B., Nascimento L.G.L., Martins E., Carvalho A.F. (2024). Immobilization of the beta-galactosidase enzyme by encapsulation in polymeric matrices for application in the dairy industry. J. Dairy Sci..

[3] Plaza-Vinuesa L., Sanchez-Arroyo A., Moreno F.J., de Las Rivas B., Munoz R. (2023). Dual 6Pbeta-Galactosidase/6Pbeta-Glucosidase GH1 Family for Lactose Metabolism in the Probiotic Bacterium *Lactiplantibacillus plantarum* WCFS1. J. Agric. Food Chem..

[4] Deng Y., Misselwitz B., Dai N., Fox M. (2015). Lactose Intolerance in Adults: Biological Mechanism and Dietary Management. Nutrients.

[5] Darma A., Sumitro K.R., Jo J., Sitorus N. (2024). Lactose Intolerance versus Cow’s Milk Allergy in Infants: A Clinical Dilemma. Nutrients.

[6] Szilagyi A., Ishayek N. (2018). Lactose Intolerance, Dairy Avoidance, and Treatment Options. Nutrients.

[7] Facioni M.S., Raspini B., Pivari F., Dogliotti E., Cena H. (2020). Nutritional management of lactose intolerance: The importance of diet and food labelling. J. Transl. Med..

[8] Swagerty D.L., Walling A.D., Klein R.M. (2002). Lactose intolerance. Am. Fam. Physician.

[9] Suez J., Zmora N., Segal E., Elinav E. (2019). The pros, cons, and many unknowns of probiotics. Nat. Med..

[10] Guarner F., Malagelada J.R. (2003). Gut flora in health and disease. Lancet.

[11] He T., Priebe M.G., Harmsen H.J., Stellaard F., Sun X., Welling G.W., Vonk R.J. (2006). Colonic fermentation may play a role in lactose intolerance in humans. J. Nutr..

[12] Hertzler S.R., Savaiano D.A. (1996). Colonic adaptation to daily lactose feeding in lactose maldigesters reduces lactose intolerance. Am. J. Clin. Nutr..

[13] Rios-Covian D., Ruas-Madiedo P., Margolles A., Gueimonde M., de Los Reyes-Gavilan C.G., Salazar N. (2016). Intestinal Short Chain Fatty Acids and their Link with Diet and Human Health. Front. Microbiol..

[14] Koh A., De Vadder F., Kovatcheva-Datchary P., Backhed F. (2016). From Dietary Fiber to Host Physiology: Short-Chain Fatty Acids as Key Bacterial Metabolites. Cell.

[15] Dunne C., O’Mahony L., Murphy L., Thornton G., Morrissey D., O’Halloran S., Feeney M., Flynn S., Fitzgerald G., Daly C. (2001). In vitro selection criteria for probiotic bacteria of human origin: Correlation with in vivo findings. Am. J. Clin. Nutr..

[16] Plaza-Diaz J., Ruiz-Ojeda F.J., Gil-Campos M., Gil A. (2019). Mechanisms of Action of Probiotics. Adv. Nutr..

[17] La Fata G., Weber P., Mohajeri M.H. (2018). Probiotics and the Gut Immune System: Indirect Regulation. Probiotics Antimicrob. Proteins.

[18] De Man J., Rogosa M., Sharpe M.E. (1960). A Medium for the Cultivation of Lactobacilli. J. Appl. Microbiol..

[19] Charteris W.P., Kelly P.M., Morelli L., Collins J.K. (1998). Antibiotic susceptibility of potentially probiotic *Lactobacillus* species. J. Food Prot..

[20] Moroni O., Kheadr E., Boutin Y., Lacroix C., Fliss I. (2006). Inactivation of adhesion and invasion of food-borne *Listeria monocytogenes* by bacteriocin-producing *Bifidobacterium* strains of human origin. Appl. Environ. Microbiol..

[21] Xue H., Zhang M., Ma J., Chen T., Wang F., Tang X. (2020). Lactose-Induced Chronic Diarrhea Results From Abnormal Luminal Microbial Fermentation and Disorder of Ion Transport in the Colon. Front. Physiol..

[22] Li Q., Wang X., Guo S., Wang T., Cao H., Cao Y., Dong B. (2025). Galacto-oligosaccharides alleviate experimental lactose intolerance associated with gut microbiota in mice. Front. Microbiol..

[23] van De Heijning B.J.M., Kegler D., Schipper L., Voogd E., Oosting A., van der Beek E.M. (2015). Acute and Chronic Effects of Dietary Lactose in Adult Rats Are not Explained by Residual Intestinal Lactase Activity. Nutrients.

[24] Moser R.L., Peo E.R., Crenshaw T.D., Cunningham P.J. (1980). Effect of dietary lactose on gain, feed conversion, blood, bone and intestinal parameters in postweaning rats and swine. J. Anim. Sci..

[25] Coutinho J., Peixoto T.S., de Menezes G.C.A., Carvalho C.R., Ogaki M.B., Gomes E.C.Q., Rosa C.A., Rosa L.H., Arantes R.M.E., Nicoli J.R. (2021). In Vitro and In Vivo Evaluation of the Probiotic Potential of Antarctic Yeasts. Probiotics Antimicrob. Proteins.

[26] Mani-Lopez E., Arrioja-Breton D., Lopez-Malo A. (2022). The impacts of antimicrobial and antifungal activity of cell-free supernatants from lactic acid bacteria in vitro and foods. Compr. Rev. Food Sci. Food Saf..

[27] Vazquez-Gutierrez P., de Wouters T., Werder J., Chassard C., Lacroix C. (2016). High Iron-Sequestrating Bifidobacteria Inhibit Enteropathogen Growth and Adhesion to Intestinal Epithelial Cells In vitro. Front. Microbiol..

[28] Gueimonde M., Jalonen L., He F., Hiramatsu M., Salminen S. (2006). Adhesion and competitive inhibition and displacement of human enteropathogens by selected lactobacilli. Food Res. Int..

[29] Rao R.K., Samak G. (2013). Protection and Restitution of Gut Barrier by Probiotics: Nutritional and Clinical Implications. Curr. Nutr. Food Sci..

[30] Hiippala K., Jouhten H., Ronkainen A., Hartikainen A., Kainulainen V., Jalanka J., Satokari R. (2018). The Potential of Gut Commensals in Reinforcing Intestinal Barrier Function and Alleviating Inflammation. Nutrients.

[31] Windey K., Houben E., Deroover L., Verbeke K. (2015). Contribution of Colonic Fermentation and Fecal Water Toxicity to the Pathophysiology of Lactose-Intolerance. Nutrients.

[32] He T., Venema K., Priebe M.G., Welling G.W., Brummer R.J., Vonk R.J. (2008). The role of colonic metabolism in lactose intolerance. Eur. J. Clin. Investig..

[33] Goh Y.J., Klaenhammer T.R. (2014). Insights into glycogen metabolism in *Lactobacillus acidophilus*: Impact on carbohydrate metabolism, stress tolerance and gut retention. Microb. Cell Fact..

[34] Kim H.S., Gilliland S.E. (1983). *Lactobacillus acidophilus* as a dietary adjunct for milk to aid lactose digestion in humans. J. Dairy Sci..

[35] Hao R., Liu Q., Wang L., Jian W., Cheng Y., Zhang Q., Hayer K., Idris R.K.R., Zhang Y., Lu H. (2023). Anti-inflammatory effect of *Lactiplantibacillus plantarum* T1 cell-free supernatants through suppression of oxidative stress and NF-κB- and MAPK-signaling pathways. Appl. Environ. Microbiol..

[36] Rezaie N., Aghamohammad S., Haj Agha Gholizadeh Khiavi E., Khatami S., Sohrabi A., Rohani M. (2024). The comparative anti-oxidant and anti-inflammatory efficacy of postbiotics and probiotics through Nrf-2 and NF-kB pathways in DSS-induced colitis model. Sci. Rep..

[37] Shen Y., Fan N., Ma S.X., Cheng X., Yang X., Wang G. (2025). Gut Microbiota Dysbiosis: Pathogenesis, Diseases, Prevention, and Therapy. MedComm.

[38] Zheng Y., Zhang Z., Tang P., Wu Y., Zhang A., Li D., Wang C.Z., Wan J.Y., Yao H., Yuan C.S. (2023). Probiotics fortify intestinal barrier function: A systematic review and meta-analysis of randomized trials. Front. Immunol..

[39] Zheng Z., Lyu W., Ren Y., Li X., Zhao S., Yang H., Xiao Y. (2021). Allobaculum Involves in the Modulation of Intestinal ANGPTLT4 Expression in Mice Treated by High-Fat Diet. Front. Nutr..

[40] Lynch J.B., Gonzalez E.L., Choy K., Faull K.F., Jewell T., Arellano A., Liang J., Yu K.B., Paramo J., Hsiao E.Y. (2023). Gut microbiota Turicibacter strains differentially modify bile acids and host lipids. Nat. Commun..

[41] Di Vincenzo F., Del Gaudio A., Petito V., Lopetuso L.R., Scaldaferri F. (2024). Gut microbiota, intestinal permeability, and systemic inflammation: A narrative review. Intern. Emerg. Med..

[42] Mafe A.N., Edo G.I., Majeed O.S., Gaaz T.S., Akpoghelie P.O., Isoje E.F., Igbuku U.A., Owheruo J.O., Opiti R.A., Garba Y. (2025). A review on probiotics and dietary bioactives: Insights on metabolic well-being, gut microbiota, and inflammatory responses. Food Chem. Adv..

